# Ancient photoreceptor shapes behavioural responses

**DOI:** 10.7554/eLife.110807

**Published:** 2026-03-04

**Authors:** N Sören Häfker

**Affiliations:** 1 Alfred Wegener Institute Helmholtz Centre for Polar and Marine Research Bremerhaven Germany

**Keywords:** non-visual light perception, non-retinal photoreceptors, rhodopsins, contrast vision, darkness detection, circadian regulation, *D. melanogaster*

## Abstract

Experiments on *Drosophila* show that an evolutionarily conserved photoreceptor that senses light outside of the retina can regulate responses to direct visual cues.

**Related research article** Kirsch V, Reinhard N, Hartlieb H, Mohr A, Rieger D, Soba P, Helfrich-Förster C, Senthilan PR. 2026. RHODOPSIN 7: An ancient non-retinal photoreceptor for contrast vision, darkness detection, and circadian regulation. *eLife* **15**:RP109811. doi: 10.7554/eLife.109811.

Our eyes are likely the most important of our sensory organs when it comes to perceiving our surroundings. However, in addition to helping us see what is happening around us, light influences us in other ways that we are not consciously aware of. Indeed, from insects and worms to birds and mammals, light receptors are not restricted to the retina of the eye: they can also be found in the brain and other parts of the body, where they engage in non-visual light perception that can affect the behaviour of organisms in a variety of ways (Mat et al., 2024).

The rhodopsins are photoreceptors – proteins from the opsin family that convert light into a cellular signal. The fruit fly, *Drosophila melanogaster*, contains seven different rhodopsins, six of which are involved in visual light-perception in the eyes. The seventh, however, is a non-visual photoreceptor that is found in the brain and the optic lobe, and is highly conserved among arthropods, such as insects, crabs, spiders and their relatives (Senthilan and Helfrich-Förster, 2016). Moreover, rhodopsin 7 (RH7) is in its function reminiscent of melanopsin, a non-visual photoreceptor that is central to the synchronization of the 24 hour circadian clock in humans and other mammals (Lucas, 2013; Panda et al., 2002). However, previous research has produced somewhat conflicting results about the functional role and location of RH7. Now, in eLife, Pingkalai Senthilan of the University of Würzburg and colleagues at Würzburg and the University of Erlangen-Nürnberg – including Valentina Kirsch as first author – report the results of experiments that shed light on the role of RH7 in *Drosophila* (Kirsch et al., 2026).

Kirsch et al. started by confirming that RH7 is expressed in neurons that form parts of the circadian clock in the central brain. (Light is able to penetrate the casing of the head in *Drosophila*, and can even penetrate the skull to reach opsins expressed in the brains of vetebrates such as birds). The researchers also confirmed that RH7 is expressed in neurons in the optic lobe, the structure that transfers information from the retina to the brain.

To explore the role of RH7 in shaping *Drosophila* behaviour, the researchers compared wild-type flies with mutant flies that lacked the gene for RH7 by exposing them to a cycle of 12 hours of light followed by 12 hours or darkness. The mutants were much less active during darkness, but were also more active during the light phase (Figure 1A). Further, mutant flies showed a clearly reduced “startle response” when they experienced a short pulse of darkness during what was otherwise a light phase (Figure 1B).

**Figure 1.**
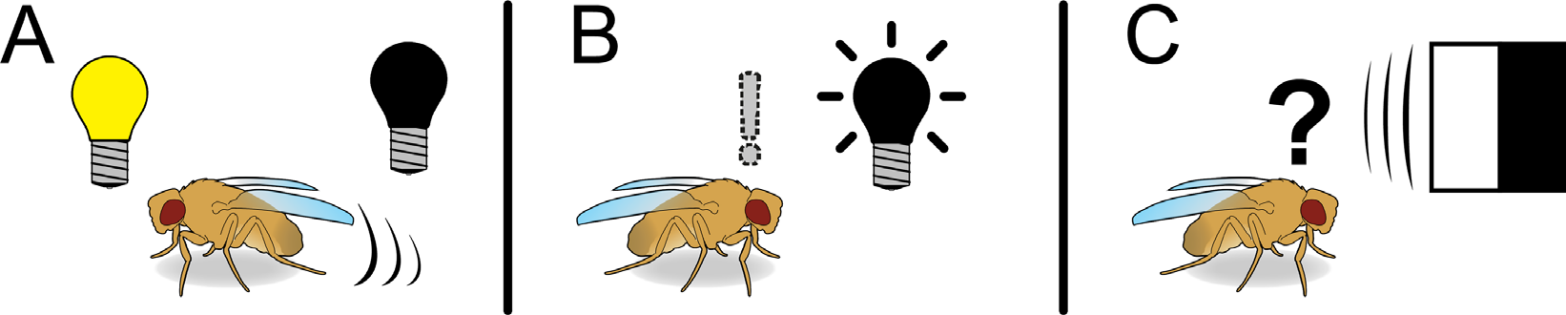
Consequences of rhodopsin 7 loss in *Drosophila melanogaster*. (**A**) Mutant flies lacking the gene for the non-visual photoreceptor rhodopsin 7 (RH7) show reduced locomotor activity during periods of darkness, and increased activity during periods of light. (**B**) Mutant flies also display a reduced “startle response”, with the size of this effect being modulated by the circadian clock. (**C**) Mutant flies further display a reduced response to moving high-contrast objects. Overall, the results of Kirsch et al. suggest that RH7 and the circadian clock work together to influence how flies respond to direct, visual cues, with the response depending on the time of day.

Although the strength of the startle response was modulated by the circadian clock over the 24 hour cycle, the clock itself was not disrupted by the loss of RH7 in the flies. This is consistent with previous studies which found that RH7 is not essential for the circadian clock, although it does influence the synchronization of the clock to day-night cycles (Kistenpfennig et al., 2017; Ni et al., 2017). Flies lacking RH7 also showed altered preferences for shade and various colours of light over the course of the day (Lazopulo et al., 2019; Meyerhof et al., 2024), with RH7 mediating the avoidance of blue light in particular. Overall, these results paint a picture of RH7 and the circadian clock working together to influence how flies respond to visual cues, with this response depending on the time of day. The loss of RH7 also led to a reduced response to moving high-contrast objects (Figure 1C), which is reminiscent of the behaviour of mammals that lack melanopsin (Sonoda et al., 2018).

Strikingly, although RH7 has been identified in various arthropods, its function has so far only been investigated in *Drosophila*. This raises the question of whether the functional role of RH7 is conserved across phylogenetic groups as well as habitats/lifestyles. Given its wide conservation and the apparent functional similarity to the mammalian non-visual photoreceptor melanopsin, there must be a clear benefit in the “context” provided by non-visual light receptors like RH7.

It will also be intriguing to disentangle how RH7 signalling modulates decision making on the neuronal and intracellular level, and achieving this will require a more detailed understanding of the cues that *Drosophila* and other species encounter in the wild. Given how old RH7 is in evolutionary terms, as well as its primary sensitivity to blue light, it could be particularly interesting to perform comparative studies with species inhabiting marine environments, where blue light dominates and circadian clocks originally evolved (Gehring and Rosbash, 2003).
